# Inhibition of tumor cell growth by adenine is mediated by apoptosis induction and cell cycle S phase arrest

**DOI:** 10.18632/oncotarget.21690

**Published:** 2017-10-09

**Authors:** Ming Han, Xin Cheng, Zhiqin Gao, Rongrong Zhao, Shizhuang Zhang

**Affiliations:** ^1^ School of Clinical Medicine, Weifang Medical University, Weifang 261053, China; ^2^ Department of Medical Imaging, Weifang Medical University, Weifang 261053, China; ^3^ School of Bioscience and Technology, Weifang Medical University, Weifang 261053, China; ^4^ Medical Imaging Center, Affiliated Hospital of Weifang Medical University, Weifang 261031, China

**Keywords:** adenine, adenosine, cell cycle, apoptosis

## Abstract

Gekko swinhonis has a long standing history in Chinese traditional medicine recognized for its application in treating patients with terminal cancer.In order to discover novel anticancer drugs with high anti-tumor efficacy and low toxicity to normal cells, we aim to investigate the anti-tumor components from Gekko swinhonis. Four nucleosides from the extracted samples were enriched, namely adenosine, guanosine, thymidine and inosine. We evaluated the antitumor effect of the four nucleosides and found that adenosine possessed the strongest anti-tumor effect. Besides, adenine could inhibit the growth of Bel-7402 and Hela cells in a dose and time dependent manner, but not normal human cervical keratinocytes. Bel-7402 and Hela cells had undergone apoptosis 48 hours after treatment as evidenced by morphologic changes under TEM, while adenine blocked cell cycle of tumor cells at S phase and subsequently causing cell cycle exit and promoting apoptosis. Moreover, the pharmacokinetics of adenine was stable in cell culture medium for up to 72 hours. Combining its potency with stability, we conclude adenine makes a promising candidate for an anti-tumor drug.

## INTRODUCTION

High anti-tumor efficacy and low toxicity to normal cells are two of the most valuable characteristics for any anticancer drug. The research and development efforts in discovering such prospects are very highly regarded in recent history. Many anti-tumor drugs have varying degrees of toxicity to hematopoietic stem cells, and are often harmful to patient’s vital organs such as heart, kidney, and liver [1]. Thus, discovering novel anticancer drug with high anti-tumor efficacy and low toxicity to normal cells is an ultimate goal for anticancer therapy.

Chinese traditional medicine has a long history of using Gekko swinhonis bundled with other herbs to treat patients with terminal diseases, such as cancer [1, 2]. However, the effective components in Gekko swinhonis that possess anti-tumor activity remain to be elucidated. Nucleosides of adenosine, guanosine, thymidine and inosine are extracted and enriched by isolating anti-tumor components from Gekko swinhonis via drug screening *in vitro*. They are common nucleosides found in eukaryotic cells. Using cancer cell lines as models, we further evaluated the antitumor effect of the nucleosides and found that adenosine possessed the strongest anti-tumor effect amongst the four. However, study has shown that adenosine released into the extracellular space is rapidly degraded by ectonucleotidases [3], which makes it an unlikely candidate for anticancer drug unless its pharmaceutics is improved to extend its stability.

Adenine is one of the two purine nucleobases present in deoxyribonucleic acid (DNA) and ribonucleic acid (RNA). It forms adenosine nucleoside when attached to ribose, and adenosine triphosphate (ATP) nucleotide when three phosphate groups are added to adenosine. ATP is basic for transferring chemical energy between bio chemical reactions. It has been well documented that purine nucleotides are now recognized as important signaling molecules, participating in a wide range of physiological and biological events [4, 5]. Studies have demonstrated that adenosine nucleoside plays important roles in regulation of cell growth and differentiation, cardiac function, inflammation, renal function and neurotransmission [6-8]. Thus, we speculate that adenine may be a potent endogenous physiologic and pharmacologic regulator of many functions in cells, including anti-tumor activity.

The common feature of nucleosides is that they are all normal substances participating in nucleic acid metabolism in eukaryotic cells. They are not considered as compounds that can be modified by transformation of the general structure to improve its pesticide effect and reduce toxicity [3]. However, according to the metabolic regularity in eukaryotic cells and chemical structure of nucleosides, studying the metabolic process of human nucleosides in tumor cells and finding the upstream and downstream molecules regulated by the nucleosides may provide insight into understanding the pathogenesis of tumors [9-11]. We have investigated 15 kinds of normal nucleoside derivatives in 4 types of primary tumor cells *in vitro*. The nucleoside derivatives include four nucleosides, four deoxynucleoside, five bases, AMP and IMP. Among the 15 nucleoside derivatives, adenosine series (adenosine, deoxyadenosine, and adenine) possesses the strongest anti-tumor activity. Since adenine has been used in the clinic as a leukocyte promoting drug for many years, we hypothesize that adenine may play an important role in metabolism of tumor cells and can eventually be developed as a viable anticancer drug. Therefore, the purpose of this study is to investigate the metabolism, pharmacodynamics and possible mechanisms of adenine’s antitumor activity, and to provide new insight into developing adenine as an anticancer drug.

## RESULTS

### The inhibitory effect of nucleosides on proliferation of Bel-7402 and Hela cells

**Figure 1:**
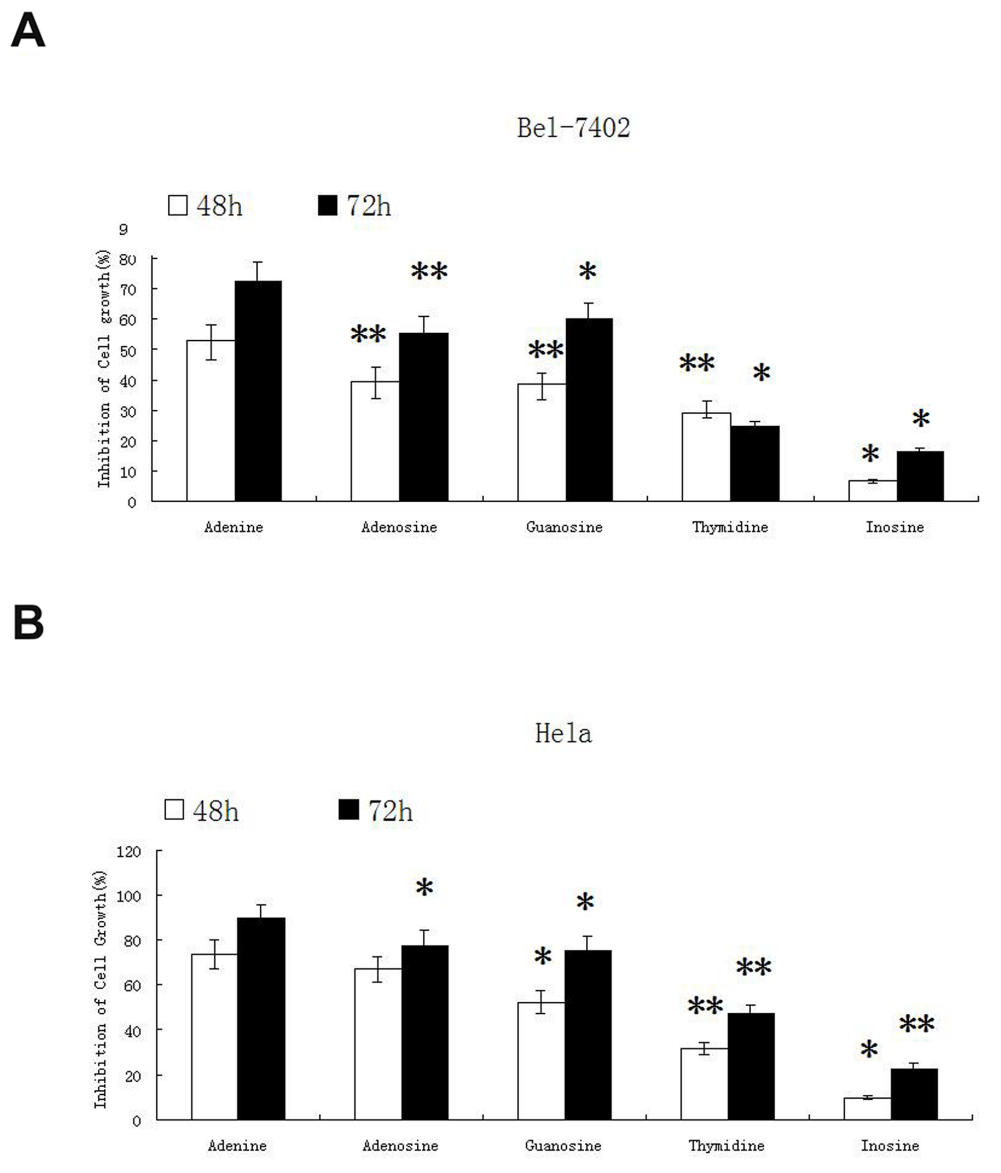
The inhibitory effect of nucleosides on proliferation of Bel-7402 and Hela cells Four nucleosides adenosine, guanosine, thymidine, inosine and adenine were tested for inhibitory effect on cancer cell lines Bel-7402 and Hela. Adenosine was used at the final concentration of 1mg/ml (3.74mM), and equal molar of the other nucleosides guanosine, thymidine, inosine and adenine (1.06mg/ml, 0.91mg/ml, 1mg/ml and 0.52mg/ml respectively) were used. The cells were treated for 48h and 72h. The inhibitory effect on cancer cell growth was evaluated by MTT assay. The experiment was repeated for three times. Statistically significant differences compared with adenine and the other nucleosides are shown. ^*^P < 0.05, ^**^P < 0.01.

The inhibitory effect of nucleosides including adenosine, guanosine, thymidine, inosine and adenine were tested on two cancer cell lines of Bel-7402 and Hela. The inhibitory effect of the other 4 nucleosides on tumor cells growth was estimated in comparison with adenosine, a known nucleotide with inhibitory effect on tumor cells. In this study, adenosine was used at the final concentration of 1mg/ml (3.74mM), and equal molar of the other 4 nucleosides including adenine, guanosine, thymidine and inosine (0.52mg/ml, 1.06mg/ml, 0.91mg/ml and 1mg/ml respectively) were used when treating cells. After 48h and 72h of treatment, the inhibitory effect of nucleosides on cancer cell growth was evaluated by MTT assay. As shown in Figure 1, adenine displayed the strongest inhibitory effect on both types of cancers. After treatment for 48h, adenine inhibited the growth of Bel-7402 cells by (53 ± 5.24)% and Hela cells by (73.6 ± 6.26)%. After treatment for 72h, adenine inhibited the growth of Bel-7402 cells by (72.6 ± 6.45)% and Hela cells by (89.5 ± 6.42)%, respectively.

### The stability of adenine in culture medium

**Figure 2:**
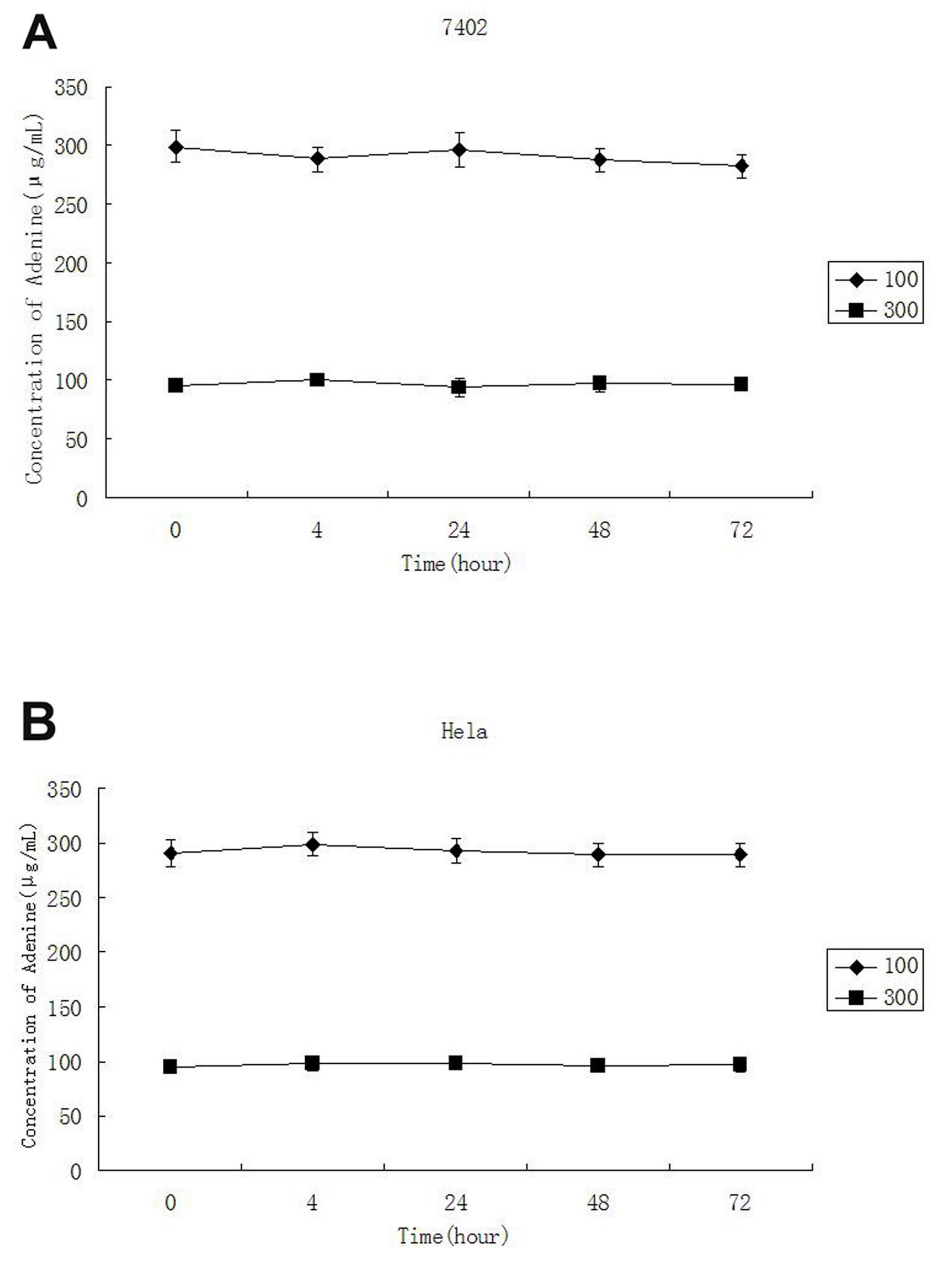
The stability of adenine in culture medium The final concentrations of adenine added in to cell culture were 300μg/ml and 100μg/ml and measure by spectrometry at 0, 4, 24, 48 and 72 hours. No significant reduction of adenine in the medium was detected between actual concentration measured and the concentration of adenine added during 72 hours incubation.

In order to determine the stability of adenine in culture medium, different concentrations of adenine were added into the cell culture medium and measured by HPLC-UV at the time point of 0, 4, 24, 48 and 72 hours. The result indicated that the concentration of adenine in the medium was slight reduced along the time. However, as shown in Table 1 and Figure 2, the concentration of adenine was not significantly reduced in the medium after incubation for 72 hours.

**Table 1:** Stability of adenine in culture medium

Time (hour)	Concentration added (μg/mL)	Concentration Measured ($\left(\bar{x}\pm S\right)$, μg/mL)
0	100	95.21±4.45
	300	299.16±12.91
4	100	99.64±5.15
	300	288.38±10.97
24	100	94.01±7.42
	300	296.44±15.11
48	100	97.12±6.84
	300	287.58±10.15
72	100	95.93±4.09
	300	282.13±9.99

### The effect of adenine on proliferation of Bel-7402 and Hela cells

**Figure 3:**
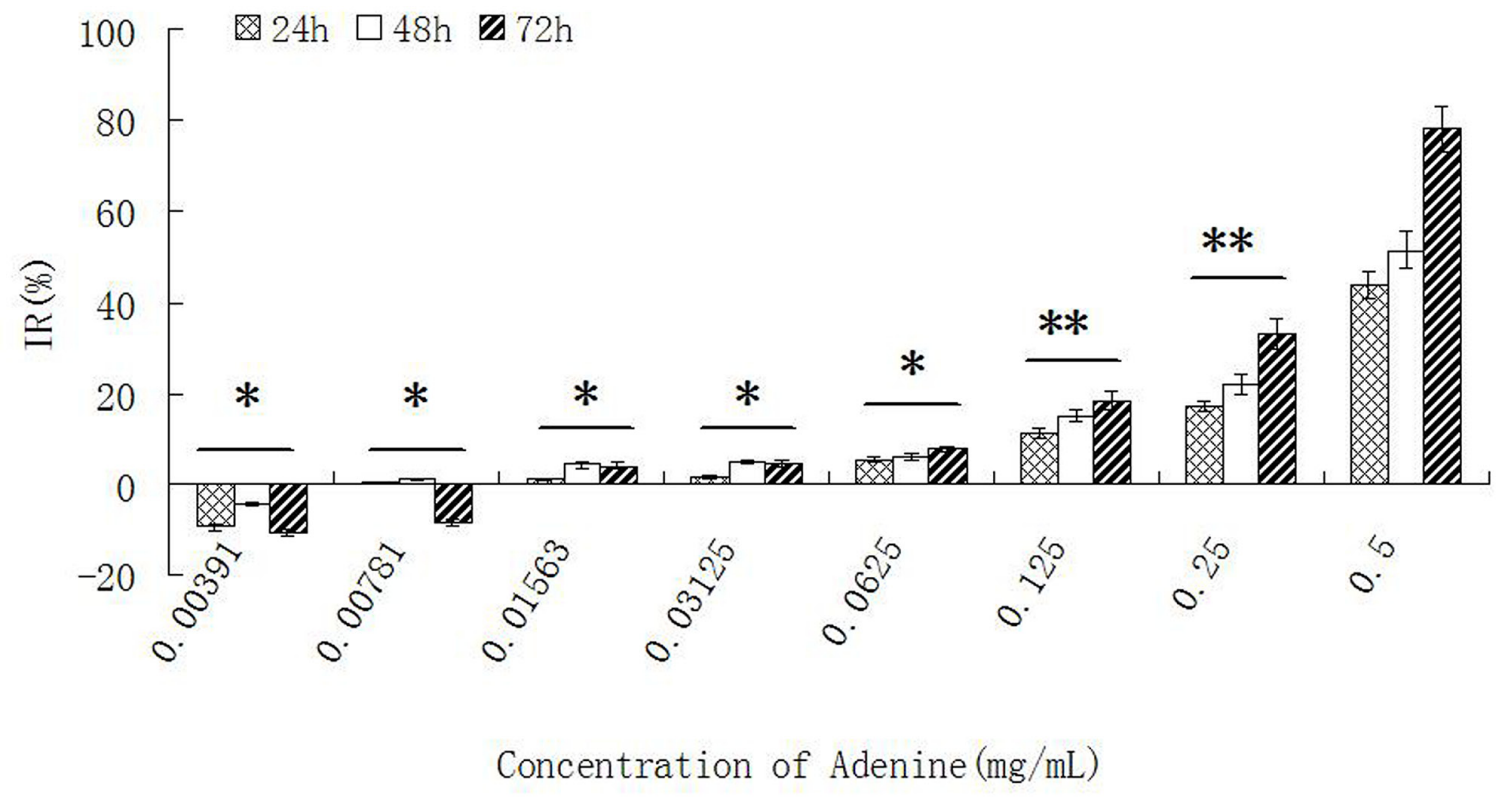
The effect of adenine on proliferation of Bel-7402 cells Bel-7402 cells were treated by adenine with different concentrations for 24h, 48h and 72h, and the inhibitory effect on the cell growth was measured by MTT assay. The inhibition of the cell growth was increased in a dose- and time- dependent manner. Statistically significant differences compared with adenine concentration of 0.5 mg/ml and other concentrations are shown. ^*^P < 0.05, ^**^P < 0.01.

The proliferation of Bel-7402 and Hela cells was inhibited when stimulated with different concentration of adenine for 24h, 48h and 72 hours. The inhibitory effect on cells growth was in a dose- and time- dependent manner. The ID_50_ of adenine at 72 hours was 0.2758 ± 0.0013 mg/ml for Bel-7402 cells, and 0.2157 ± 0.0009 mg/ml for Hela cells. The result was shown in Table 2 and Figure 3.

**Table 2:** Effect of adenine on the proliferation of Bel-7402 cells

Groups	Concentration (mg/ml)	24h		48h		72h	
		OD	IR(%)	OD	IR(%)	OD	IR(%)
Control	-	0.335±0.013	-	0.355±0.014	-	0.313±0.010	-
	0.00391	0.367±0.017	-9.51±0.79	0.371±0.020	-4.50±0.65	0.347±0.011	-10.89±0.89
	0.00781	0.334±0.014	0.36±0.01	0.351±0.012	1.11±0.12	0.340±0.008	-8.51±0.81
	0.01563	0.331±0.011	1.12±0.12	0.341±0.010	4.15±0.46	0.301±0.007	4.13±0.45
	0.03125	0.329±0.008	1.57±0.19	0.338±0.013	4.83±0.53	0.299±0.005^*^	4.55±0.51
Adenine	0.06250	0.317±0.012	5.42±0.69	0.334±0.009^*^	5.96±0.52	0.289±0.006^**^	7.76±0.69
	0.12500	0.298±0.017^**^	11.16±0.97	0.302±0.007^**^	14.91±1.33	0.256±0.008^**^	18.22±1.86
	0.25000	0.254±0.016^**^	17.19±1.11	0.277±0.011^**^	22.15±2.21	0.210±0.005^**^	32.94±3.24
	0.50000	0.189±0.009^**^	43.68±3.01	0.172±0.008^**^	51.42±4.13	0.060±0.007^**^	78.18±5.21

^*^compared with control *P*<0.05; ^**^compared with control *P*<0.01

After treatment Bel-7402 and Hela cells with different concentration of adenine, the shrinkage of tumor cells were observed and the percentage of adherent cells was decreased estimated by morphology. In contrast to the control group, circular transparent particles were appeared in the cytoplasm of tumor cells and nuclear fission was reduced. Apoptotic cells were increased. These morphological changes were in dose- and time- dependent manner.

### The effect of adenine on proliferation of normal cervical cells

**Figure 4:**
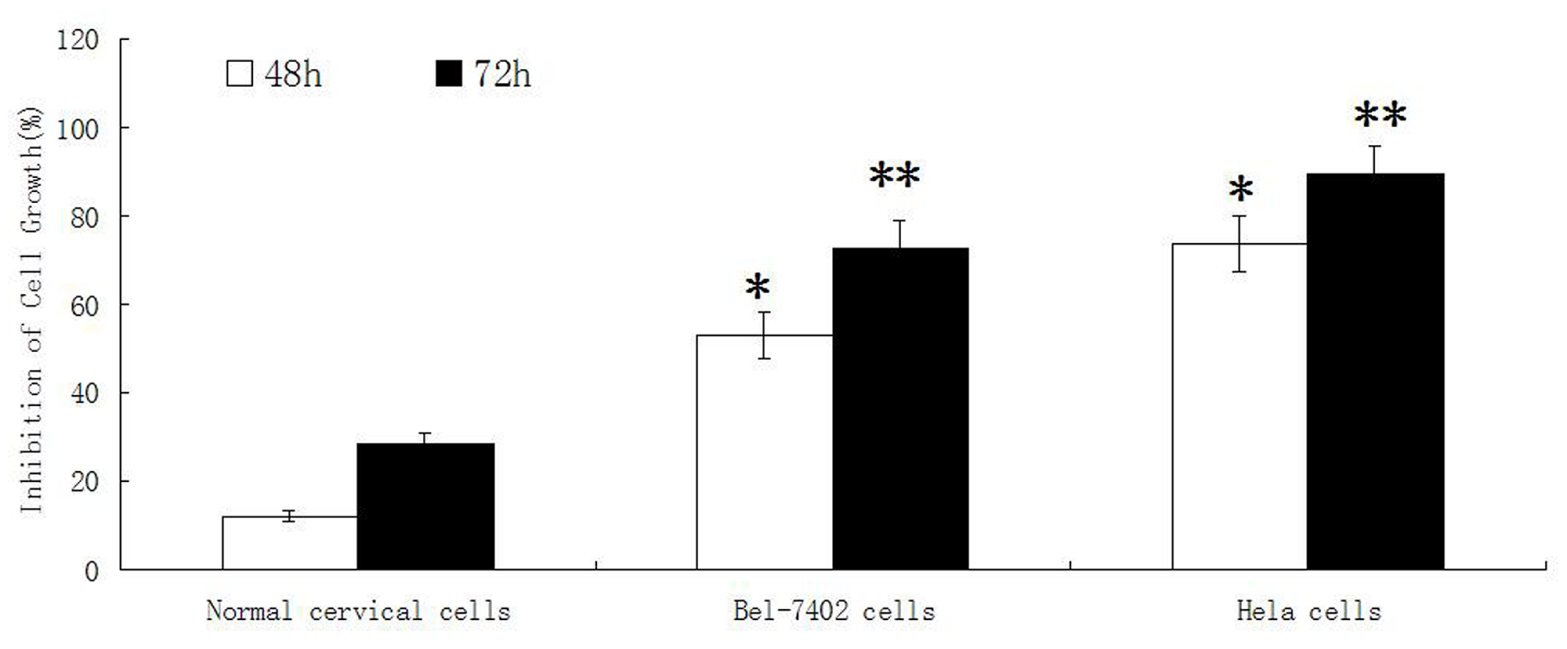
The effect of adenine on proliferation of normal cervical cells, Bel-7402 cells and Hela cells Normal cervical cells, Bel-7402 cells and Hela cells were treated by adenine at the concentration of 0.5mg/ml for 48h and 72h, The inhibitory effect of adenine on the cell growth was measured by MTT assay. Adenine displays a much weaker inhibitory effect on normal cervical cell than that on Bel-7402 cells and Hela cells. ^*^P < 0.05, ^**^P < 0.01.

The proliferation of normal cervical cells was inhibited when treated with different concentrations of adenine for 24h, 48h and 72 hours. With the increase concentration and time of adenine stimulation, the inhibitory effect on cells growth was increased slightly. The ID_50_ of adenine at 72 hours was 0.6027 ± 0.0158 mg/ml. As shown in Figure 4, after treatment for 48h and 72h, adenine displayed a much weaker inhibitory effect on normal cervical cells than cancer cells of Bel-7402 and Hela.

### The ultrastructural changes of Bel-7402 and Hela cells treated with adenine

**Figure 5:**
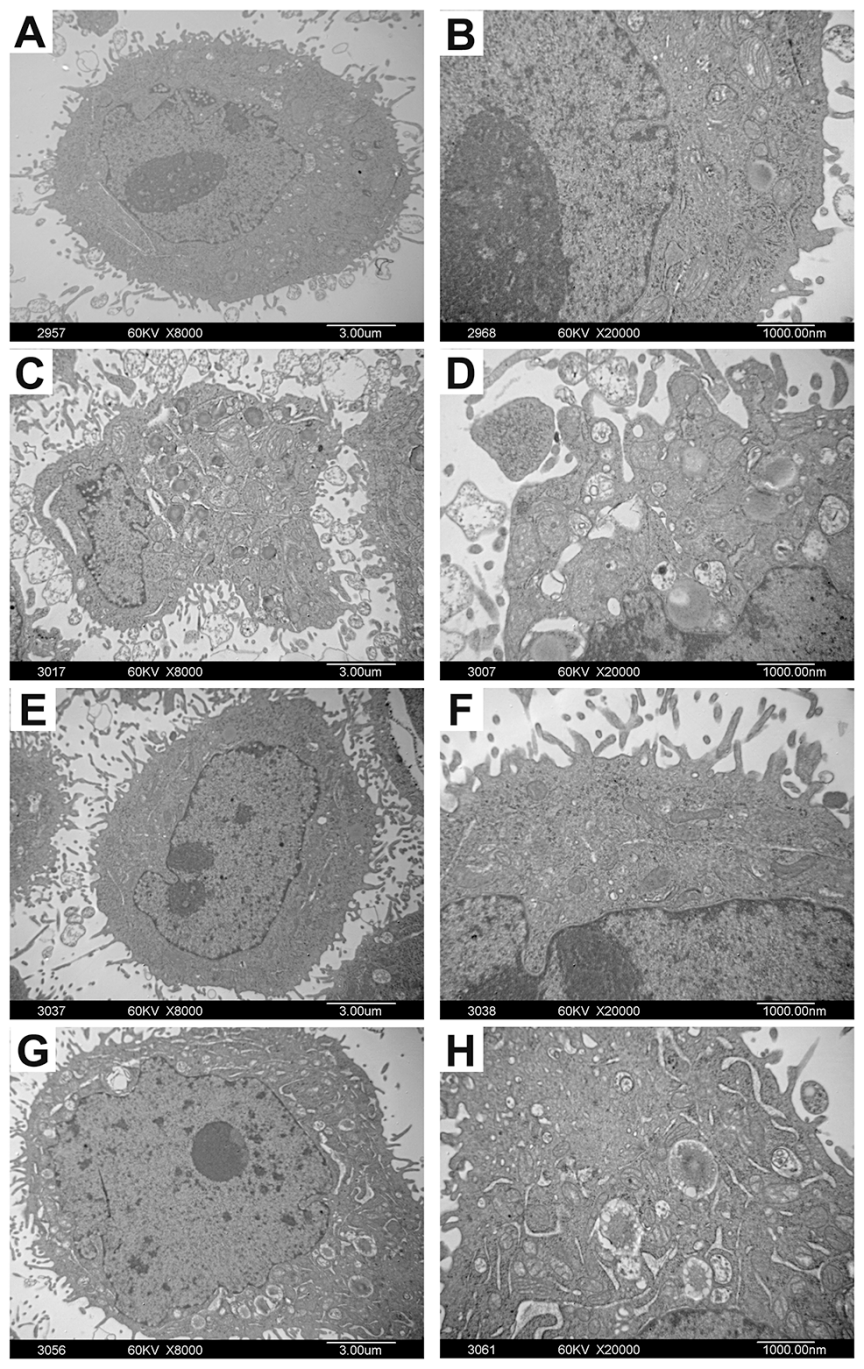
The ultrastructural changes of Bel-7402 and Hela cells treated with adenine Bel-7402 and Hela cells were treated with adenine (0.5mg/ml) for 48 hours. Figure **(A, B)** are control cells without adenine treatment. Figure **(C-H)** are cells treated with adenine. Under TEM, the cells treated with adenine show increase of nuclear heterochromatin, shrinkage and condensation of the nuclear membrane edge, irregular shaped nucleus and nuclear fragmentation (C, D); Increase of Microvilli on the cell surface (E, F); Increase of cytoplasmic vacuolization, mitochondria swelling and vacuolar degeneration. Rough endoplasmic reticulum became thickened and widened and ribosome de-granulated (G, H).

Under TEM, the ultra-structure of Bel-7402 and Hela cells was displayed clearly. The nuclear chromatin distribution was uniform and rich mitochondria were observed. The surface of the cells was with many microvilli and pseudopodia projections. As shown in Figure 5, after treatment with adenine for 48h, nuclear heterochromatin was increased in cells. Shrinkage and condensation of the nuclear membrane was observed as a well-circumscribed mass or crescent. The nucleus became in irregular shapes or even nuclear fragmentation. Microvilli on the cell surface, cytoplasmic vacuolization, mitochondria intact, mitochondria swelling and vacuolar degeneration were most obvious in cells. Rough endoplasmic reticulum became thickened and widened, and ribosome was degranulated.

### The effects of adenine on cell cycle of Bel-7402 and Hela cells

**Figure 6:**
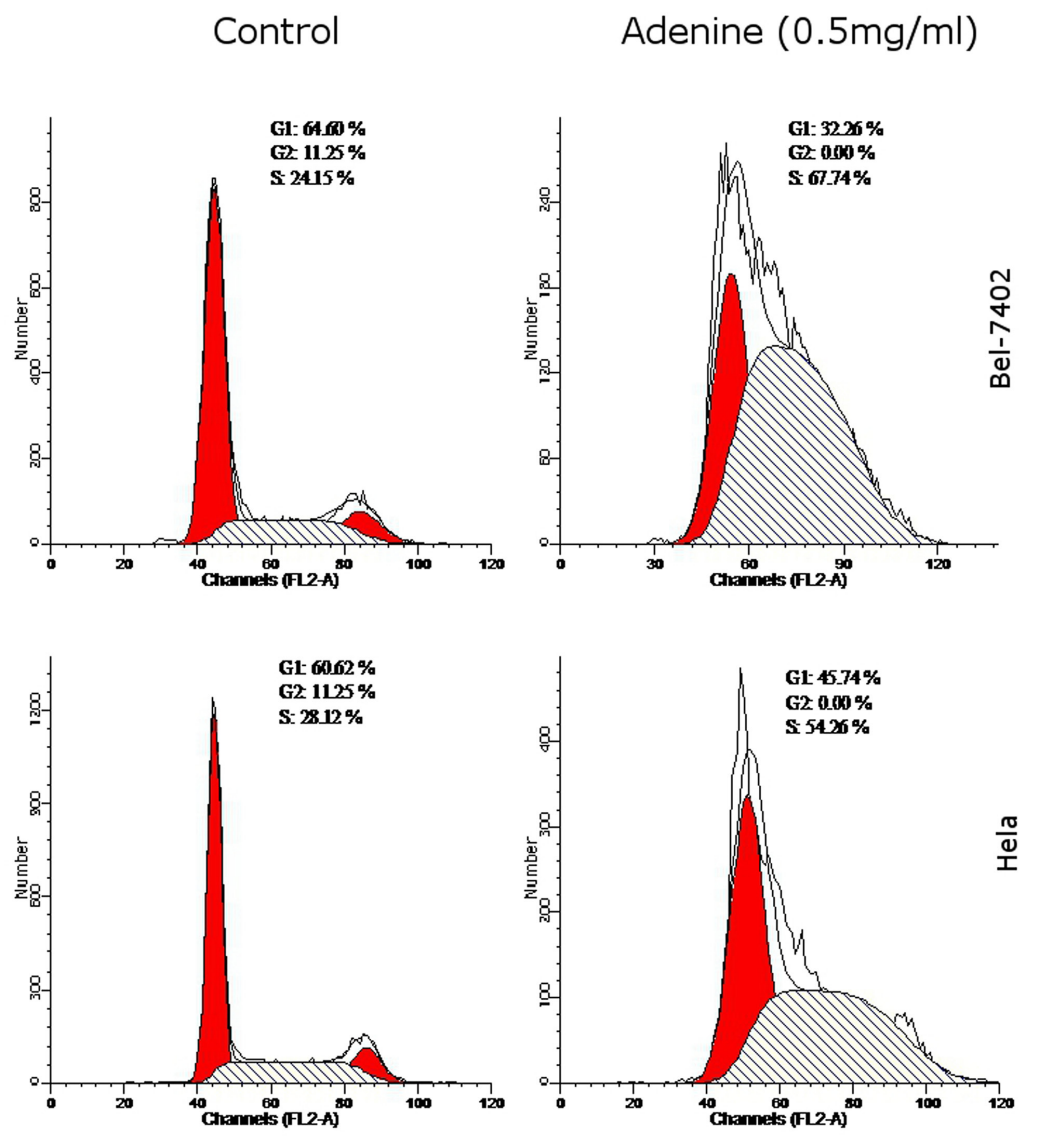
The effects of adenine on cell cycle of Bel-7402 and Hela cells Bel-7402 cells (upper panel) and Hela cells (lower panel) were treated with adenine (0.5mg/ml) for 24h, and cell cycle was analyzed by flow cytometry using PI staining. Left panel represents the control cells without treatment; right panel represents the cells treated with adenine. The percentage of cell cycle G1, G2 and S phases were indicated in each figure.

As shown in Table 3, compared with controls, G0/G1 phases were significantly reduced by (32.26 ± 1.74)%, S phase was increased by (67.74 ± 1.93)% and G2/M phases were significantly reduced by (0.01 ± 0.00)% in Bel-7402 cells. In Hela cells, G0/G1 phases were reduced by (45.74 ± 1.38)%, S phase was increased by (54.26 ± 1.56)% and G2/M phases were significant reduced by (0.01 ± 0.00)% compared with control. The findings suggested that adenine caused S phase arrest in tumor cells (Figure 6).

**Table 3:** Effect of Adenine on the cell cycle of Bel-7402 cells and Hela cells ($\left(\bar{x}\pm S\right)$, n=3)

Cells	Group	G1(%)	S(%)	G2(%)
Bel-7402 cells	control	64.60±1.61	24.15±1.34	11.25±1.12
	adenine	32.26±1.74^**^	67.74±1.93^**^	0.01±0.00^**^
Hela cells	control	59.61±1.79	30.15±1.36	10.24±1.18
	adenine	45.74±1.38^*^	54.26±1.56^**^	0.01±0.00^**^

^*^compared with control *P*<0.05; ^**^compared with control *P* < 0.01

## DISCUSSION

By isolating and purifying the active components from Gecko Swinhonis, we obtain four active monomers, namely adenosine, guanosine, thymidine and inosin. The four active monomers isolated from Gecko Swinhonis are all normal substances participating in nucleic acid metabolism in eukaryotic cells. We have analyzed the nucleoside metabolic process and their upstream and downstream compounds, and inferred that nucleosides and their normal derivatives might have anti-tumor effects. By investigating the anti-tumor effect of 15 nucleoside derivatives in 4 primary tumor cells, we have found that adenosine, deoxyadenosine and adenine have the strongest anti-tumor effects, while adenine has slightly better anti-tumor effect *in vitro* than adenosine at equal molar concentrations.

Adenine, molecular formula C_5_H_5_N_5_ and CAS number 73-24-5, has many functions in biochemistry. It forms adenosine triphosphate (ATP), nicotinamide-adenine dinucleotide (NAD), and flavin-adenine dinucleotide (FAD) in cell respiration. Besides, adenine is a nuclear base in composition of nucleic acid (DNA or RNA). Adenine is a vital purine that is generated *in vivo* by two pathways including the de novo purine synthesis and the salvage pathway mediated through the degradation of 5’-deoxy-5’-methylthioadenosine (MTA) [12]. The enzyme methyl thioadenosine phosphorylase (MTAP) converts MTA, which is generated during the synthesis of polyamines, directly into adenine and 5-methylthioribose-1-phosphate [13]. Interestingly, loss of MTAP activity or deletion of the MTAP gene has been reported to occur in a variety of primary tumors, such as acute lymphoblastic leukemia [14], non-small-cell lung cancer [15], and melanoma [16]. Therefore, study of MTAP may bring new insight to understanding the tumorigenesis and tumor-selective therapy. For several decades, adenine has been used as a drug to increase leukocytes and can be administrated orally, intramuscularly or intravenously. Therefore, adenine is a promising anti-tumor drug.

The stability of a drug *in vitro* is important so that it can be developed into an anti-tumor drug. Some anti-tumor drugs may metabolize rapidly, however, their anti-tumor activity may be attributed to their secondary metabolites. Our data have implicated that adenine is stable in culture medium and keeps its anti-tumor activity for more than 72 hours. Thus, the anti-tumor effect of adenine is likely self-induced and does not require any secondary components to function.

Uncontrolled cell growth and reduced apoptosis are important characteristics of malignancies. One of the most important parameter for screening anti-tumor drug is that the drug has inhibitory effect on tumor cell proliferation and minimal toxicity to normal cells. Previous studies have shown that adenosine and AMP inhibited cell proliferation in a wide range of tumor cells [17, 18]. Our preliminary study has suggested that active components isolated from Gecko Swinhonis possessed inhibitory effects on cancer cells growth and induction of apoptosis in a panel of cancer cell lines, including cell lines of Bel-7402, HepG-2, lung cancer cell lines 95-C, Hela, and Bcap-37. In the present study, we further demonstrated that adenine possessed strong inhibitory effect on the growth of cancer cells of Bel-7402 and Hela. Besides, the inhibitory effect was in a dose and time dependent manner. Compared with adenosine, adenine showed stronger inhibitory effect on the growth of Bel-7402 and Hela cells. Furthermore, we found that the anti-tumor effect of adenine was closely related to the growth status of tumor cells, namely, the faster tumor cells proliferated, the stronger the inhibitory effect of the adenine had on the cells. We also found that the anti-tumor effect of adenine was slightly stronger than adenosine at equal molar concentration.

Although we found the anti-tumor effect of adenine, mechanisms of adenine in tumorigenesis and its difference with other nucleoside regarding the anti-tumor activity remain largely unknown. Aberrant growth of tumor cells is primarily attributed to reduced apoptosis and aberrant cell cycle regulation. Previous studies have demonstrated that adenosine was capable of inhibiting cancer cells growth by inducing apoptosis [9, 19]. It has been suggested that aberrant adenosine metabolism may be associated with aberrant growth of cancer cells [20]. Accordingly, identifying new drugs to regulate cell cycle and induce cell apoptosis is essential for clinical application. In the present study, the effect of the adenine on the apoptosis and cell cycle of cancer cells was estimated. In the first stage of apoptosis, the morphological changes in cell surface were limited to reduction of some specialized structures such as microvillus, whereas the cell membrane remained intact. The mitochondria in cytoplasm remained intact, ribosome gradually breaked down and the endoplasmic reticulum capsular space expanded and gradually fused with the plasma membrane. In addition, chromatin was condensed and often formed a crescent-shaped structure. The second stage featured as the formation of apoptotic bodies. The nuclear chromatin was fragmentized in different sizes and gathered with certain organelles such as mitochondria, and many vesicular bud bubbly protrusions emerged at the cell surface and formed apoptotic bodies. The morphological features of tumor cells treated by adenine complied with the characteristics of apoptosis. Given unbalanced apoptosis process is one of the most important mechanisms for tumor formation and growth, induced apoptosis of tumor cells by adenine makes it a potent anti-tumor drug. The mechanism that is involved in the anti-tumor effect of adenine has not been elucidated yet, although our data suggests that adenine promotes apoptosis of cancer cells. More functional studies are warranted for further investigation.

Cell cycle is a basic process of cells movement and an inherent mechanism of eukaryotic cells with self-replicating and high conservation. Many anti-tumor drug kill or inhibit tumor cells grow by blocking cell cycle progression. Therefore, cell cycle is an important target of cancer therapy via blocking cell cycle progression and promoting apoptosis of tumor cells. Many anticancer drugs can block the cell cycle at the G1 or G2/M phases. Similar to findings in the study by Hatse et al. [6], we found that adenine increased the proportion of cells in S phase and reduced the proportion of cells in G2/M phases. Adenine blocked tumor cells in the S phase, and leaded to tumor cell proliferation cycle arrest and subsequently apoptosis.

In summary, the present study demonstrates that anti-tumor effect of adenine is stable up to 72 hours. Adenine can significantly inhibit the proliferation of Bel-7402 and Hela cells in a dose- and time-dependent manner. In addition, adenine can block the cell cycle of tumor cells at S phase and subsequently cause cell cycle exit and thus promote tumor cells apoptosis. Therefore, Adenine is a promising candidate for an useful anti-tumor drug.

## MATERIALS AND METHODS

### Cancer cell lines and cell culture

Human hepatocellular carcinoma cell line Bel-7402 and human cervical cancer cell line Hela were purchased from the Cell Bank of Chinese Academy of Sciences (Beijing, China). Cells were cultured with RPMI-1640 medium (Gibco, Grand Island, USA), supplemented with 10% FCS (Solarbio, Beijing, China) and 3000 U/ml penicillin-streptomycin (Gibco, BRL MD, USA) in an incubator at 37°C with 5% CO_2_ in air. The cells growing in logarithmic growth phase were used for experiments.

### Culture of normal human cervical cells

Normal human cervical epithelial cells were obtained from hysterectomy specimens of healthy women. Tissue fragments were incubated with 0.25% trypsin (Gibco BRL, MD, USA) for 24 hours at 4°C. The growth medium was Keratinocyte-SFM (Gibco, Grand Island, USA), supplemented with 5% FCS, 1 μg/ml of fungizone (Gibco BRL, MD, USA) and 3000 U/ml penicillin-streptomycin (Gibco BRL, MD, USA). Subconfluent cultures were dispersed with 0.0025% trypsin and 0.02% EDTA. Local ethical approval was obtained before commencing this study and as appropriate, tissue was collected with informed consent.

### Stability of adenine in culture medium

The Bel-7402 and Hela cells were cultured with RPMI-1640 medium, supplemented with 10% FCS and antibiotics in an incubator at 37°C with 5% CO_2_ in air at the concentration of 3.5×10^5^ cells/ml. For experiment, the cells were divided into three groups: cells treated with 300μg/ml of adenine, cells treated with 100μg/ml of adenine and cells untreated as control. After treatment, cells were harvested for experiments at the time points of 0h, 4h, 24h, 48h, and 72h.

### MTT assay

100μL of the cell suspension containing 2.0×10^3^ cells was added to each well in 96-well plate and cultured for 24 h at 37°C. After a serial dilution, adenine was added to the cell culture with the final concentrations of 0.5, 0.25, 0.125, 0.0625, 0.03125, 0.01563, 0.00781 and 0.00391 mg/ml. The cell morphology and the number were evaluated at 0h, 24h, 48h and 72h. To evaluate the cell growth, 20μL of MTT (Amresco, Ohio, USA) at the concentration of 5mg/ml was added into each well in 96-well plate, and cells were continuously incubated for an additional 4 hours. After carefully removing the supernatant, 150μL of DMSO (Solarbio, Beijing, China) was added to each well and the plate was shaken genteelly until DMSO was completely dissolved. Optical density values of every well were measured at 570 nm by microplate reader. Inhibition ratio and 50% inhibiting concentration of adenine on the tumor cell growth were calculated. The experiments were repeated for three times.

### Ultrastructural analysis of Bel-7402 and Hela cells treated by adenine

1.0 × 10^6^ Bel-7402 or Hela cells in logarithmic phase were cultured at 37°C with 5% CO_2_ in air for 24h and then adenine was added to cell culture at the final concentration of 0.5mg/ml. Control group were only cultured with RPMI-1640 medium containing 10% FCS. After 48h incubation, the cells were harvested and fixed with 2.5% glutaraldehyde for 4h, followed by 1% osmic acid fixation at 4°C for 1h. The cells were dehydrated with ethanol and acetone, and the cells were embedded in epoxy resin. Ultrathin section of the samples was made and subsequently stained. Ultrastructure of cells was observed by transmission electron microscopy (TEM).

### Cell cycle analysis of Bel-7402 and Hela cells treated by adenine

Bel-7402 cells and Hela cells growing in logarithmic phase were treated with a final concentration of 0.5mg/ml of adenine. After 24h, the cells were harvested, washed by PBS and fixed with pre-cold 75% ethanol for 2 hours at 4°C. After two washes with PBS, the cells were incubated with RNase A at the final concentration of 100μg/ml for 30min. The cells were then stained with PI at the final concentration of 50 ug/ml for 15 minutes at room temperature. Cell cycle was analyzed using flow cytometry (FACS Caliper, DB, USA).

### Statistical analysis

Statistical analysis was performed with SPSS 19.0 software. Student’s *t*-test (two-tailed distribution with a two sample equal variance) was applied for analysis. All results were presented as the mean ± SD. *P* < 0.05 was considered to be statistically significant.

## References

[1] Chen D, Yao WJ, Zhang XL, Han XQ, Qu XY, Ka WB, Sun DG, Wu XZ, Wen ZY (2010). Effects of Gekko sulfated polysaccharide-protein complex on human hepatoma SMMC-7721 cells: inhibition of proliferation and migration. J Ethnopharmacol.

[2] Zhang SX, Zhu C, Ba Y, Chen D, Zhou XL, Cao R, Wang LP, Ren Y, Wu XZ (2012). Gekko-sulfated glycopeptide inhibits tumor angiogenesis by targeting basic fibroblast growth factor. J Biol Chem.

[3] Zimmermann H, Braun N (1999). Ecto-nucleotidases—molecular structures, catalytic properties, and functional roles in the nervous system. Prog Brain Res.

[4] Robak P, Robak T (2013). Older and new purine nucleoside analogs for patients with acute leukemias. Cancer Treat Rev.

[5] Yang Z, Cheng W, Hong L, Chen W, Wang Y, Lin S, Han J, Zhou H, Gu J (2007). Adenine nucleotide (ADP/ATP) translocase 3 participates in the tumor necrosis factor induced apoptosis of MCF-7 cells. Mol Biol Cell.

[6] Hatse S, Schols D, De Clercq E, Balzarini J (1999). 9-(2-Phosphonylmethoxyethyl) adenine induces tumor cell differentiation or cell death by blocking cell cycle progression through the S phase. Cell Growth Differ.

[7] Saito M, Yaguchi T, Yasuda Y, Nakano T, Nishizaki T (2010). Adenosine suppresses CW2 human colonic cancer growth by inducing apoptosis via A1 adenosine receptors. Cancer Lett.

[8] Ralevic V, Burnstock G (1998). Receptors for purines and pyrimidines. Pharmacol Rev.

[9] Gallerne C, Touat Z, Chen ZX, Martel C, Mayola E, Sharaf el dein O, Buron N, Le Bras M, Jacotot E, Borgne-Sanchez A, Lemoine A, Lemaire C, Pervaiz S, Brenner C (2010). The fourth isoform of the adenine nucleotide translocator inhibits mitochondrial apoptosis in cancer cells. Int J Biochem Cell Biol.

[10] Terry KL, De Vivo I, Titus-Ernstoff L, Shih MC, Cramer DW (2005). Androgen receptor cytosine, adenine, guanine repeats, and haplotypes in relation to ovarian cancer risk. Cancer Res.

[11] Yalowitz JA, Pankiewicz K, Patterson SE, Jayaram HN (2002). Cytotoxicity and cellular differentiation activity of methylenebis (phosphonate) analogs of tiazofurin and mycophenolic acid adenine dinucleotide in human cancer cell lines. Cancer Lett.

[12] Shi J, Liu HF, Wong JM, Huang RN, Jones E, Carlson TJ (2011). Development of a robust and sensitive LC–MS/MS method for the determination of adenine in plasma of different species and its application to *in vivo* studies. J Pharm Biomed Anal.

[13] Pegg AE, Williams-Ashman HG (1969). Phosphate-stimulated breakdown of 5'-methylthioadenosine by rat ventral prostate. Biochem J.

[14] Batova A, Diccianni MB, Nobori T, Vu T, Yu J, Bridgeman L, Yu AL (1996). Frequent deletion in the methylthioadenosine phosphorylase gene in T-cell acute lymphoblastic leukemia: strategies for enzyme-targeted therapy. Blood.

[15] Olopade OI, Buchhagen DL, Malik K, Sherman J, Nobori T, Bader S, Nau MM, Gazdar AF, Minna JD, Diaz MO (1993). Homozygous loss of the interferon genes defines the critical region on 9p that is deleted in lung cancers. Cancer Res.

[16] Fitchen JH, Riscoe MK, Dana BW, Lawrence HJ, Ferro AJ (1986). Methylthioadenosine phosphorylase deficiency in human leukemias and solid tumors. Cancer Res.

[17] Ohkubo S, Nagata K, Nakahata N (2007). Adenosine uptake-dependent C6 cell growth inhibition. Eur J of Pharmacol.

[18] Saitoha M, Nagaia K, Nakagawab K, Yamamurab T, Yamamotoa T, Nishizaki T (2004). Adenosine induces apoptosis in the human gastric cancer cells via an intrinsic pathway relevant to activation of AMP-activated protein kinase. Biochem Pharmacol.

[19] Brenner C, Subramaniam K, Pertuiset C, Pervaiz S (2011). Adenine nucleotide translocase family: four isoforms for apoptosis modulation in cancer. Oncogene.

[20] Mujoomdara M, Hoskin D, Blay J (2003). Adenosine stimulation of the proliferation of colorectal carcinoma cell lines: Roles of cell density and adenosine metabolism. Biochem Pharmacol.

